# Analyzing Social Media to Infer Mental Health Status and Affective States for Crisis and Disaster Management: Scoping Review

**DOI:** 10.2196/79762

**Published:** 2026-07-20

**Authors:** Francesca Müller, Samuel Tomczyk, Frank Fiedrich

**Affiliations:** 1 Chair for Public Safety and Emergency Management, Faculty of Mechanical Engineering and Safety Engineering, University of Wuppertal, Wuppertal, Germany; 2 Department Health and Prevention, Institute of Psychology, Universität Greifswald, Robert-Blum-Straße 13, Greifswald, 17489, Germany, 49 038344203806; 3 Partner Site Greifswald/Rostock, German Center for Child and Adolescent Health (DZKJ), Greifswald, Germany; 4 Institute and Policlinic of Medical Psychology and Medical Sociology, Universitätsmedizin Rostock, Rostock, Germany

**Keywords:** social media analysis, mental health, crisis management, disaster response, decision-making, situational awareness, passive data collection, psychosocial needs

## Abstract

**Background:**

The use of social media (SoMe) during crisis and disaster situations (CaDs) has gained increasing attention across disciplines. However, existing research is highly fragmented and often focused on technical aspects, with a limited understanding of how and which psychosocial information is derived from SoMe in CaDs.

**Objective:**

This scoping review provides an overview of the current research landscape regarding the analysis of SoMe data during CaDs to obtain information about public mental health and psychosocial needs. It identifies key themes, methodological approaches, and research gaps, with a particular focus on relevance for the German context.

**Methods:**

Following a scoping review protocol, a structured database search was conducted in PubMed, Web of Science, and Scopus to identify peer-reviewed studies published up to 2025. A method of triangulation combining qualitative and quantitative approaches was applied. The studies were analyzed regarding the type of CaDs, geographical focus, classification systems, methods of analysis used, and inclusion of psychosocial aspects (such as affect and mental health status).

**Results:**

Overall, we identified 179 studies that examined 267 CaDs. Of the included studies, 76% (136/179) focused on natural disasters, with biological CaDs representing 23% (41/179) of these events. For Germany, 5 studies were found, with only one covering storms, floods, or extreme temperatures, despite these making up most of the disasters in Germany per EM-DAT (Emergency Events Database) data. Most studies used datasets from Asia (especially China), while Africa was examined less often, pointing to differences in geographical representativeness. To infer mental health status or affective state, 47 studies used machine learning, 87 studies used lexicon-based approaches, and 25 studies used a combination; 14 studies used manual coding, and few studies did not explicitly mention their approach. Mental health outcomes ranged from affective valence (positive, negative, or neutral) to specific primary (eg, fear) and secondary (eg, denial) emotions and needs (eg, resources). Yet, few studies were based on theoretical models or included end-user perspectives. No study conducted real-time analysis; instead, all were retrospective. Additionally, current research focuses primarily on deficits (eg, psychological needs, negative affect, or stress), and often neglects positive mental health outcomes (eg, resilience and collective coping).

**Conclusions:**

This scoping review underlines the rising popularity of SoMe analysis in CaDs regarding public mental health and needs. Although different techniques were developed and tested, there remain major gaps in real-time application, end-user integration, and contextual adaptation—particularly for underrepresented regions such as Africa, but also in countries such as Germany. As most models were developed or tested retrospectively (eg, using data from the COVID-19 pandemic), future research should examine the validity and tenability of such models in real-time monitoring and data, and emphasize more user-centered design and participatory research, theoretical grounding, and practical utility.

## Introduction

### Rationale

An accurate assessment of a population’s psychological and social needs and resources is of importance in the initial phases of crisis and disaster situations (CaDs), as it significantly influences situational awareness, operational planning, and ultimately the success of response efforts [1]. This assessment follows decision-making processes understood as iterative cycles of reflection and action, structured in phases of situation assessment, planning, execution, and control [2-4]. Particularly in CaDs, decision-making becomes complex due to time pressure, the scale of affected populations, high uncertainty, and the multiplicity of actors involved [5,6]. To support this complexity, leadership structures such as crisis management teams are established, relying on interdisciplinary coordination and shared situational awareness [2,7,8]. These teams jointly analyze CaDs, develop action plans (eg, regarding technical assistance and medical support), and implement, evaluate, and adapt these plans.

In recent years, so-called psychosocial situational awareness has received increased attention. It refers to psychological and social aspects of affected populations that influence their capacity for adaptive or maladaptive responses in CaDs, their readiness to implement actions, but also their psychological and social states as a consequence of CaDs. Thus, in addition to technical and event-related assessments, situational awareness should also consider psychosocial aspects, such as behaviors, perceptions, and emotional states of the affected populations. In Germany, this is conceptualized as the so-called Lagebild Bevölkerungsverhalten (population behavior situation picture), which addresses psychological and social processes such as risk perception, emotional responses (eg, fear, grief, and anger [9]), trust in authorities, and individual resilience capacities [10-12]. This understanding contributes to forecasting behavioral responses and informs crisis communication and resource planning [9,13]. Notably, the psychosocial situation picture—as part of the population behavior situation picture at the municipal, operational-tactical level—supports the identification of needs, available resources, and the self-help capacities of affected individuals and communities [11,14-16].

Given the increasing relevance of digital communication in daily life, social media (SoMe) have emerged as critical platforms for the detection of real-time psychosocial signals in CaDs, as well. These platforms constitute dynamic, nonphysical spaces shaped by social interaction, where emotional content circulates and influences public behavior [17-19]. Digital monitoring, processing, and showcasing of such content—referred to as psychosocial digital situation picture—can provide valuable insights into emotional trends, mental well-being, and community needs [20-22]. From a crisis informatics perspective, SoMe are useful for active and passive information gathering in CaDs, due to their accessible and scalable sociality, networked interactions, and multimodal content [23-25].

**Figure 1.**
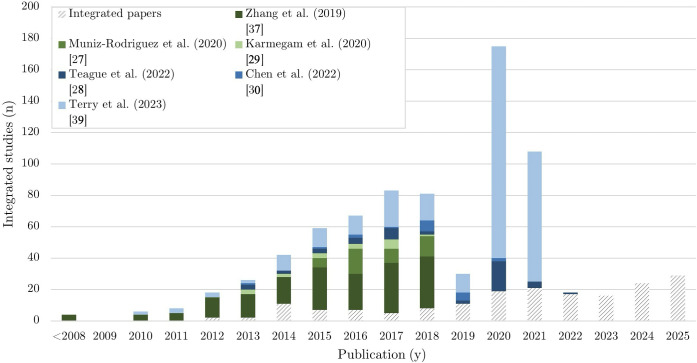
Number of unique and overlapping included studies in the scoping review compared to previous reviews on social media analytics in crisis and disasters; the stacked bar chart illustrates the number of included papers across the different reviews over time. No relevant publications were identified in any of the reviewed studies for the years 2005 to 2007 and 2009. The observed differences in paper selection are due to slightly varying research focuses and the associated use of different search terms. The hatched area indicates the number of papers uniquely included in the present scoping review.

Generating and monitoring a psychosocial digital situation picture based on SoMe use in CaDs can therefore help to support and initiate community coping mechanisms as well as facilitate informed decision-making in crisis and disaster management by either providing support or integrating self-help capacities of the population based on the information derived from SoMe. This is also a unique opportunity to address misinformation or uncertainty via SoMe communication in times of the infodemic [26] by correcting misconceptions and distributing verified reports. However, there are meaningful differences regarding the type of information and informational value for disaster management. While population behaviors can often be directly derived from active (eg, planned or reported actions in SoMe posts) or passive (eg, type of platform, duration, and interactions) SoMe use data, affective states and mental health are more challenging to analyze. There is some active communication about mental health (eg, explicit statements of stress, relief, or anger), but because mental health status can be associated with shame and stigma and also impair information processing and communication skills, it is important to consider passive SoMe use data and paraverbal indicators of communication. The research in this area has also evolved in recent years, but was often focused on specific disasters or disorders and thus does not capture the similarities and differences across disasters, methods, and states (Figure 1 [27-32]). Given the pronounced heterogeneity of the research field, the extensive data volume, and the diversity of study types and methodological approaches, a scoping review was deemed the most appropriate methodological framework for this investigation [33].

### Objectives

This review aims to conduct a scoping review of the interdisciplinary literature providing an overview of the use of collected SoMe data in the general context of CaDs to identify the mental health and affective state of the population, with a focus on methods, types of focused content, and practical inference. Thus, our research objectives include assessing the following research questions.

RQ1: What methodological approaches for analyzing SoMe data are documented in current research literature to infer mental health and affective states of populations during CaDs?

RQ2: Which dimensions of mental health and affective states are extracted from SoMe data during CaDs according to existing studies, and how are these conceptualized in the literature?

RQ3: What practical implications for crisis and disaster management can be synthesized from the existing body of research on SoMe analytics of mental health during CaDs?

### Distinction From Existing Reviews

The analysis of SoMe data in CaDs spans multiple disciplines, including communication studies, psychology, computer science, and sociology, resulting in a highly diverse body of literature. This scoping review distinguishes itself from previous work by focusing specifically on the psychosocial dimension and mental health, adopting a broader CaDs scope without restricting event types or time frames [27-30,34-36]. Unlike prior reviews, which often target narrower topics such as warning dissemination [27] or specific disorders [28], this review integrates diverse methodological approaches to highlight their relevance for crisis management, and thus includes a larger number of primary studies (Figure 1). Synthesizing fragmented findings across fields can be derived from SoMe in CaDs. A detailed overview of the differentiation compared to reviews can be found in Multimedia Appendix 1 [27-30,34,35,37-39] (Section S1.1).

## Methods

### Study Design

The review follows the JBI (Joanna Briggs Institute) methodology [40] and is reported in accordance with the PRISMA-S (Preferred Reporting Items for Systematic Reviews and Meta-Analyses literature search extension) [41] and PRISMA-ScR (Preferred Reporting Items for Systematic Reviews and Meta-Analyses extension for Scoping Reviews) checklist [31], as detailed in Checklists1  2. Relevant studies were identified through a systematic database search using elaborated Boolean search strings, complemented by backward and forward citation tracking [32]. In line with the exploratory nature of the scoping method, we did not perform a quality or risk of bias appraisal due to the heterogeneity of study types. Instead, the analysis focused on the frequency distribution of key study characteristics, content-based synthesis of findings, and identification of research gaps. The following sections outline the distinction from existing reviews, search strategy, inclusion and exclusion criteria, and the data extraction process.

### Search Strategy

To explore the use of SoMe data to infer mental health status and psychological states during CaDs, three core categories were defined to guide the search strategy: SoMe, CaDs, and psychosocial needs and resources. Following the approach of Nordhausen and Hirt [42], keywords and synonyms for each category were combined using Boolean operators across selected databases. Specific SoMe platforms (eg, X [formerly known as Twitter; X Corp], WhatsApp [Meta], Instagram [Meta], or TikTok [ByteDance]) were included as search terms to capture studies that explicitly refer to these services. As many platform names are not consistently represented in controlled vocabularies such as MeSH (Medical Subject Headings), these terms were primarily used as free-text search terms. The search strategy combined field-restricted keyword searches with controlled vocabulary terms where available. During pilot searches, including the abstract field substantially increased the number of records with only marginal relevance to the research question. To balance sensitivity and specificity of the search strategy, several key terms were therefore restricted to the title field (and author keywords where available), ensuring that retrieved papers had a primary focus on the intersection of SoMe, crisis events, and mental health. The final search strings, optimized iteratively based on preliminary results, are documented in Section S1.1 in Multimedia Appendix 2.

### Information Sources

Searches were conducted in three multidisciplinary databases, namely PubMed, Web of Science, and Scopus, which cover medicine, psychology, communication, and social sciences in both English and German. Searches were performed until November 27, 2025, across all three platforms. In addition, we also searched reference lists of previously published reviews and meta-analyses on similar topics (ie, snowballing method), and additional manual searches, clinical registries, or conference proceedings were not considered. The earliest included paper was published in 2012. Although scoping reviews may consider gray literature, such sources were excluded due to the broad and complex scope of the topic, to maintain feasibility.

### Selection of Sources of Evidence

All identified records were systematically documented in Microsoft Excel and screened in a three-stage process: first, records were retrieved based on database searches and reference lists of other reviews, and duplicates were removed; second, titles and abstracts were screened; and third, full texts screened for eligibility. To ensure intercoder reliability, a second reviewer independently screened a random sample of 10% (18/179) of the studies identified through the search string.

### Eligibility Criteria for the Inclusion and Exclusion of Studies

This scoping review focuses on peer-reviewed scientific publications that analyze data from SoMe platforms to assess psychological or psychosocial factors in the context of CaDs. Eligible studies include (1) papers addressing any type of CaDs (eg, man-made), (2) without restrictions regarding publication date, type of study (except for commentaries, opinion pieces, or editorials), or geographic setting. Exclusion criteria comprise studies that (1) use SoMe solely as a communication tool (eg, for participant recruitment), (2) focus primarily on political or marketing objectives, or (3) investigate the effects of general SoMe use in the population without relation to CaDs. Additionally, (4) publications addressing individual psychological crises without a broader social or collective CaD framework, as well as (5) those centered on medical public health topics or (6) organizational reputation in CaDs, were not considered. These criteria were defined to identify studies that relate to crisis and disaster management and promise implications for this field. Finally, we did not include (7) abstracts, reports, or conference proceedings that did not provide a full description of the study (eg, a conference poster without additional explanation) or were not peer-reviewed. An overview of all inclusion and exclusion criteria is provided in Section S1.2 in Multimedia Appendix 2.

### Data Items

Data extraction tables were developed using Microsoft Excel. For each paper that met the inclusion criteria, specific characteristics were extracted and documented based on the full-text analysis. The extracted information included (1) general information (title, authors, year, language, and research objective); (2) details of the crisis or disaster (year, type, name, location, and time frame); (3) relevant insights for decision-makers (implications for decision-makers, communicators, and data analysts; types of visualizations used); (4) data collection procedures (platforms, data acquisition methods: self-monitoring vs datasets, time periods, variables used such as keywords, accounts, or locations, and tools applied); (5) characteristics of the analyzed dataset (volume, language, data format, and integration of crisis management cycle phases); (6) analytical procedures (manual vs technical analysis, development vs adaptation vs application of existing algorithms, focus of analysis, category development, methodology, tools, and metrics); and (7) identified categories of mental health and psychosocial needs and resources, and identified research gaps.

A more detailed description of these characteristics is available in Section S1.3 in Multimedia Appendix 3. The data extraction tables were defined by the research team a priori based on internal discussions and expert recommendations.

### Data Charting Process and Synthesis of Results

The data analysis was conducted using quantitative and qualitative techniques. To reduce the complexity of the textual information and generate meaningful, abstracted insights, the method of summarizing qualitative content analysis by Mayring and Fenzl [43] was applied following a deductive-inductive approach. For this purpose, key information was extracted from the papers in accordance with the category system built on the literature and a priori defined paper characteristics, such as general information, sample characteristics, or types of examined disasters [44,45], which was refined during the analysis (Multimedia Appendix 3). For example, recommendations addressing decision-makers (eg, explicit references to “emergency management agencies,” “decision-makers,” “policy-makers,” and “governmental actors”) were extracted from the papers and compiled. These statements were then inductively analyzed and synthesized, including paraphrasing, generalization, inductive category formation, and iterative revision of the category system. This method allows for the preservation of the core meaning of original statements while producing a condensed representation of essential findings.

Furthermore, a descriptive quantitative analysis of relevant data from the full-text papers was performed (eg, types of CaDs and analysis techniques). Overall, in line with the reporting of a scoping review, the results are presented in a narrative synthesis.

## Results

### General Overview

**Figure 2.**
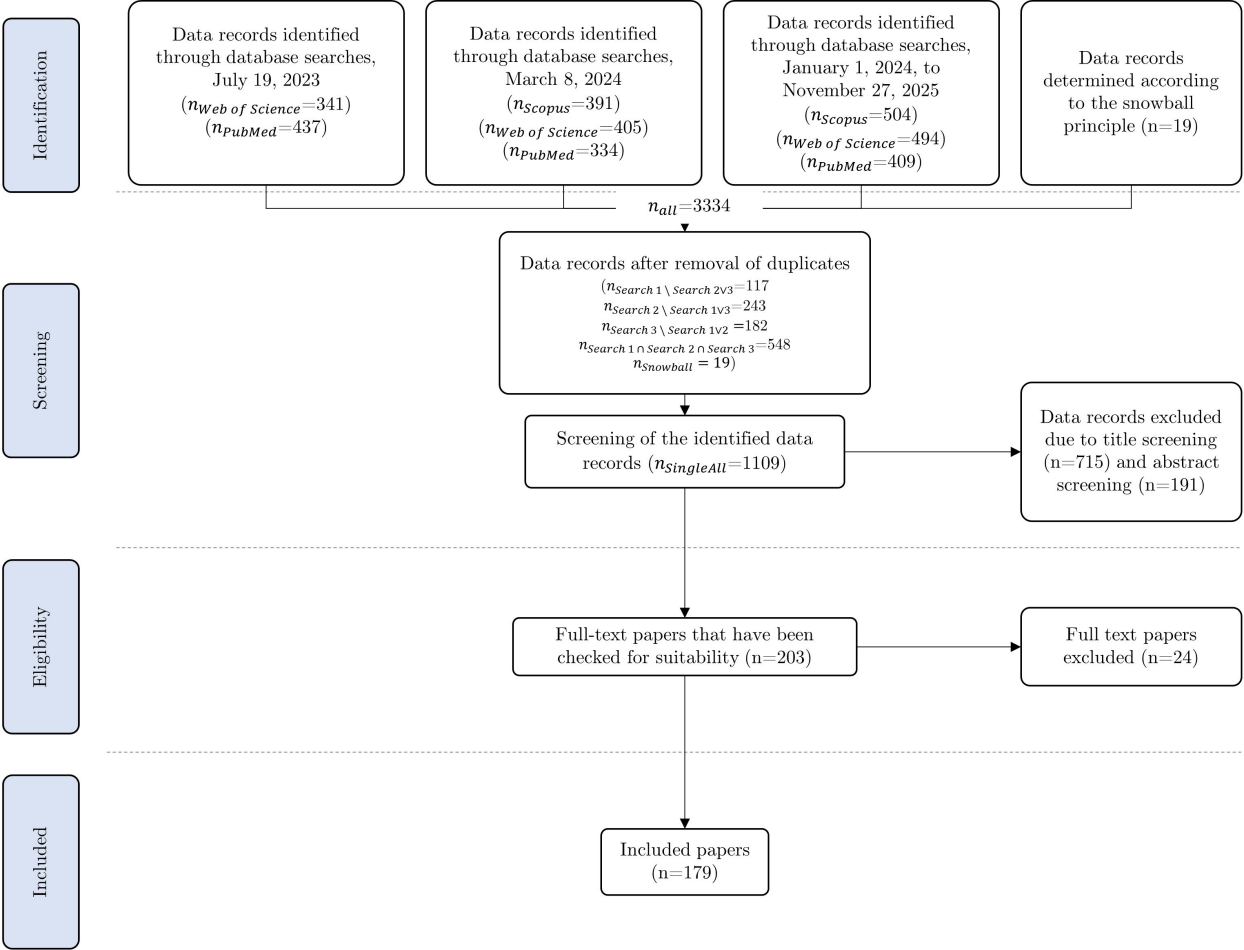
Flow diagram of the PRISMA-ScR in the scoping review (n=179); the systematic literature selection process was conducted and documented in accordance with the PRISMA guidelines. PRISMA: Preferred Reporting Items for Systematic Reviews and Meta-Analyses; PRISMA-ScR: Preferred Reporting Items for Systematic Reviews and Meta-Analyses extension for Scoping Reviews.

In total, 1727 records were retrieved (Figure 2), including 1 German-language paper, which was excluded at the full-text stage. After removing duplicates using Excel, 1109 unique papers remained for screening. Title- and keyword-based screening led to the exclusion of irrelevant studies, while abstracts were assessed when relevance was unclear. Records without abstracts were retained for full-text review. The final set included 203 potentially relevant full texts (including 19 added through backward citation search according to Wohlin et al [32]), of which 179 papers met all inclusion criteria and were included in the data extraction phase.

The reporting of the results is structured according to the research questions and divided into four sections: (1) characteristics of the included studies, (2) applied methods for inferring mental health, (3) the type of content related to mental health observed on SoMe, and (4) implications for SoMe analytics in CaD management.

### Characteristics of the Included Studies

#### Examined Crises and Disasters

Most included studies focused on the acute phase of CaDs and examined expression of psychosocial states during and immediately after an event (within a few hours or days of the event) on SoMe. They mostly used lexicon-based analysis methods and concentrated on emotions and sentiment underlying SoMe messages, such as basic emotions or valence (positive, negative, or neutral) based on linguistic inquiry (see Table 1 below).

**Table 1.** Top 10 most commonly used models in the included studies.

Model	Number of studies	Studies
Lexicon-based linguistic models		
LDA^a^	23	[46-69]
VADER^b^	19	[7,46,52,54,64,65,67,68,70-80]
LIWC^c^	18	[12,19,61,72,81-94]
SentiStrength^d^	11	[49,87,95-103]
Machine learning		
SVM^e^	23	[7,20,50,64,79,97,100,103-118]
NB^f^	15	[20,79,96,109,113,116-125]
BERT^g^	12	[7,61,69,110,117,124,126-131]
RF^h^	10	[50,55,59,103,109,115,117,124,132]
Other		
Manual	14	[133-146]
Unspecified	14	[56,105,122,147-158]

a LDA: latent Dirichlet allocation.

b VADER: Valence Aware Dictionary and Sentiment Reasoner.

c LIWC: Linguistic Inquiry Word Count.

d SentiStrength: sentiment strength detection for short informational text.

e SVM: support vector machine.

f NB: naïve Bayes text classification.

g BERT: bidirectional encoder representations from transformers.

h RF: random forest algorithm.

The papers were published between 2012 and 2025. The 179 included studies examined a total of 267 CaDs. Meteorological CaDs (57/267, 21%), including Hurricane Sandy, Hurricane Harvey, and Hurricane Irma, and biological CaDs (63/267, 23%, including the COVID-19 pandemic, H1N1, Ebola, and EHEC [enterohemorrhagic *Escherichia coli*]) were the most frequently studied events. Human-made intentional CaD (38/267, 14%, including terrorist attacks, mass shootings, and war) publications typically analyzed CaDs that occurred 3.3 years before publication (SD 2.2 y, range 0‐19 y). Data predominantly came from acute phases of CaDs. In 26% (47/179) of studies, data spanned precrisis, crisis, and postcrisis phases. All studies conducted analyses on complete datasets rather than sequentially.

**Figure 3.**
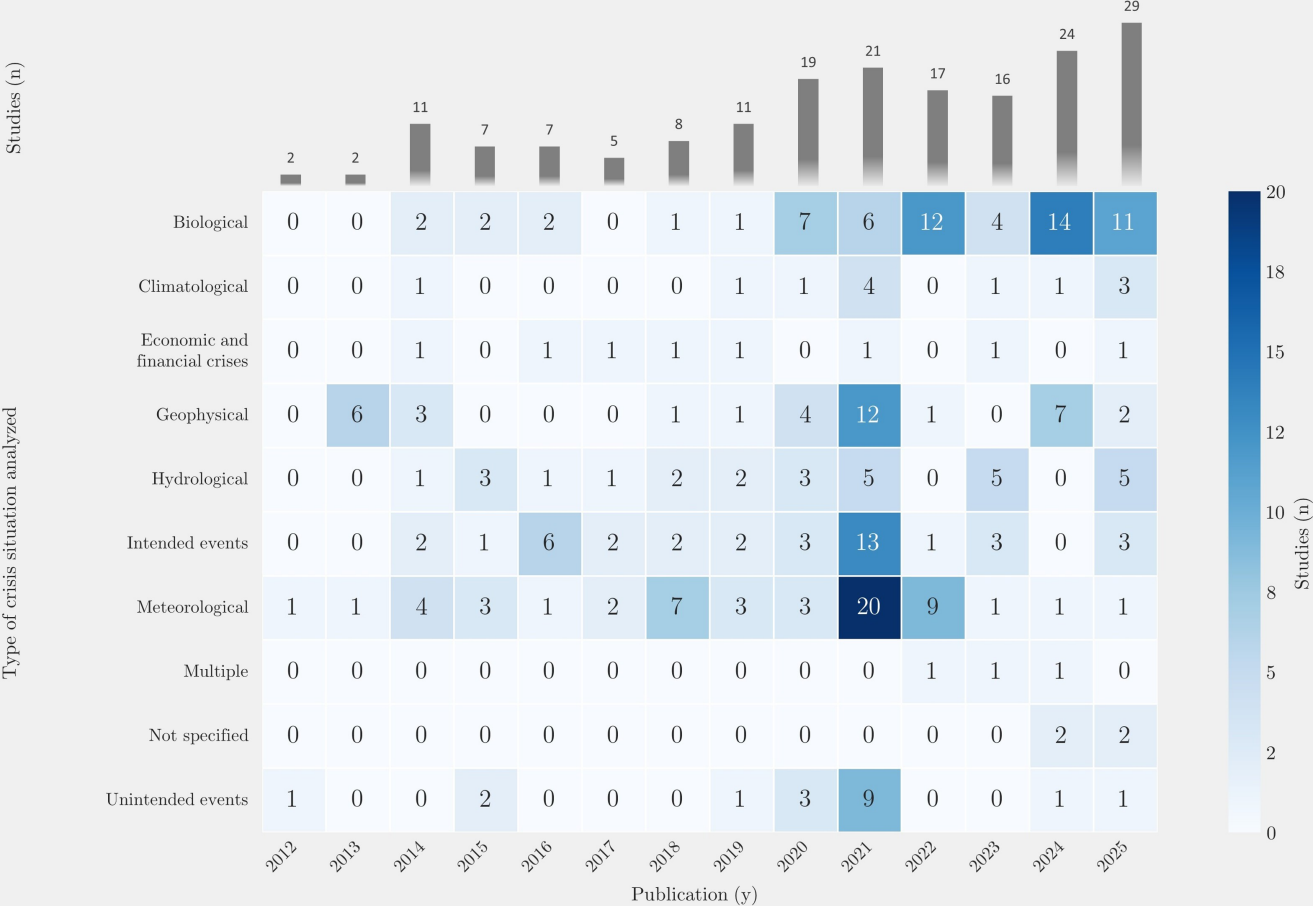
Number of studies by year of publication and type of analyzed crises and disasters; the heatmap displays the number of analyzed crisis and disaster types per publication year (total n=267), using both color intensity and absolute frequency to visualize the data. Additionally, the bar chart above the heatmap shows the absolute number of included papers per publication year (total n=179 included studies).

Figure 3 presents a matrix visualization showing the frequency of studies in relation to publication year and type of CaDs analyzed. This visualization reveals that peaks in publication activity occurred in 2021 and 2025 (driven by the COVID-19 pandemic). For the same reason, biological CaDs dominated research focus beginning in 2020, becoming the most investigated hazard category in 2022. The visualization also highlights further emphasis on meteorological CaDs in the literature.

The most frequently examined single CaDs were the COVID-19 pandemic, followed by Hurricane Sandy. A total of 4 studies did not clearly specify the CaDs examined. In most cases, the exact locations of the analyzed CaDs were not identified (49/267, 18%). Where specified, CaDs in the United States were the most frequently reported, followed by multinational events, China, and India. From a continental perspective, most studies examined CaDs in Asia (72/267, 27%) and the Americas (61/267, 23%). Additionally, 5 studies used data from CaDs in Germany (only 2 of which included German-language SoMe data). These focused on (1) Münster van attack on April 7, 2018 [159,160], (2) G20 Summit in Hamburg on July 7-8, 2017 [159,160], (3) sexual assaults during New Year’s Eve 2015 in Cologne [81], (4) outbreak of EHEC O104 in Germany in 2011 [133], and (5) Ahr valley flood in 2021 [161].

Additionally, 9 studies incorporated German SoMe data in their analysis, 7 of which included it as one of multiple languages [46,81,95,126,133,162-165]. The most frequently examined SoMe platforms were X, Sina Weibo (Weibo Corporation), and two different platforms (eg, combinations of X and Sina Weibo for data detection). Nearly all papers specified the underlying data volume (174/179, 97%), with a range of 492 to 310,000,000 posts per study.

#### Methods for Inferring Mental Health Status and Affective States From SoMe in Crisis and Disasters (RQ1)

**Figure 4.**
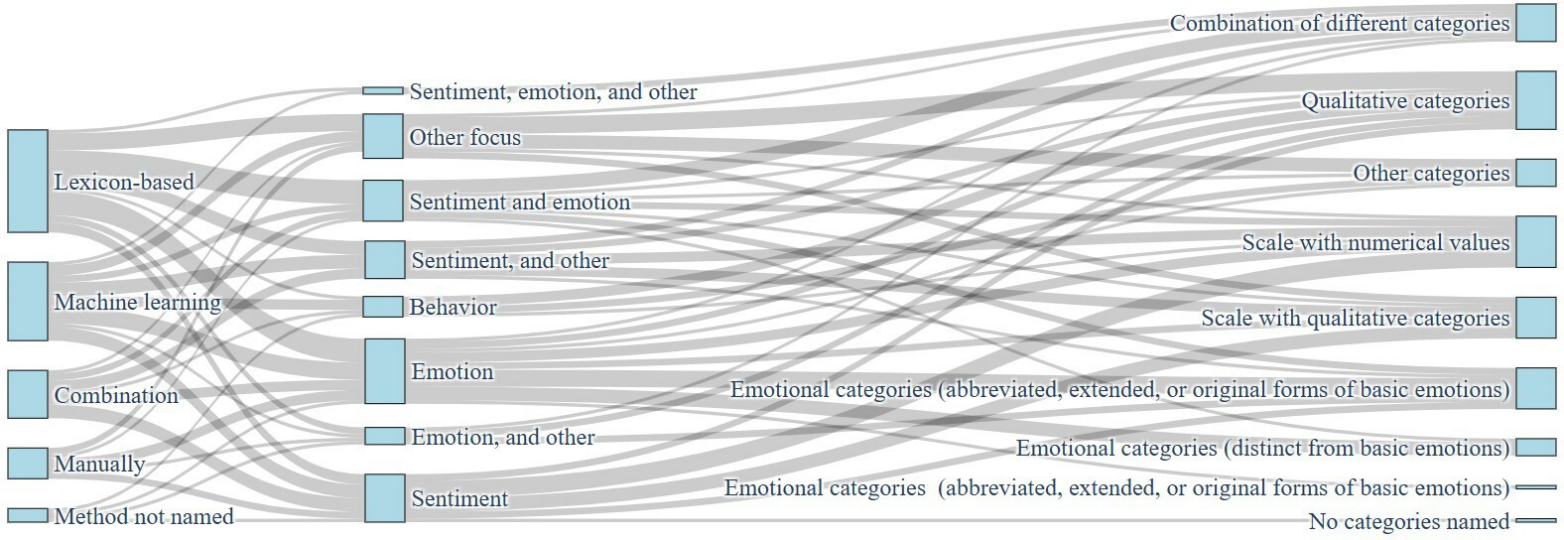
Alluvial diagram illustrating the information processing: techniques, focus topics, and classifications in the analysis of social media use in crisis and disasters; the area size of each entity represents the proportion of studies assigned to it. The width of the connecting flows indicates the share of each entity represented within the linked categories. The abbreviations used refer to lexicon-based, ML, and combination (combined use of lexicon-based and ML). ML: machine learning.

The alluvial diagram presented in Figure 4 illustrates the analysis approaches found in the reviewed literature. On the left side, it shows four main methodological categories: lexicon-based linguistic methods, machine learning (ML) methods, a combination of ML and lexicon-based methods, and manual data analysis.

In the center of the diagram, these methods branch into subcategories representing the specific psychosocial analytical focus, such as “sentiment and emotions,” “sentiment and other aspects,” or “behavior.” The streams culminate on the right in various classification schemes, ranging from binary classifications to extended models of basic emotions. For instance, the category “scale with numerical values” typically refers to continuous measures (eg, from −1 to +1), “scale with qualitative categories” reflects distinctions such as positive, neutral, and negative, while “basic emotions” denotes the use of models such as that of Plutchik [166] or Ekman [167], in either reduced or extended forms. The multiplicity of flows in the diagram highlights the variability of methodological applications to data analysis. Moreover, the strength of the connections and the size of the entities indicate that: (1) lexicon-based methods were used most frequently, (2) lexicon-based and ML methods were rarely combined, and (3) each methodological approach was mainly applied to the dominant focus areas of “sentiment” and/or “emotion.”

Table 1 summarizes the ten most frequently used data analysis models in the included studies. Among the evaluated methods, latent Dirichlet allocation (LDA) and support vector machines perform equally well and jointly lead the list, with other lexicon-based approaches such as VADER (Valence Aware Dictionary and Sentiment Reasoner) following closely. Since late 2024, the adoption of ML-based techniques, including large language models, has increased markedly. In many studies, ML approaches are used either in comparison with or in combination with lexicon-based methods. Despite lexicon-based approaches remaining dominant across the entire dataset, the results indicate a clear upward trend in the use of ML techniques. Many studies also report self-developed models or adaptations of existing models.

While lexicon-based tools were often used to identify specific emotions or valence of SoMe data, ML-based approaches were also used to explore more complex topics, such as the identification of needs.

#### Dimensions of Mental Health and Affective States Derived From SoMe During Crises and Disasters (RQ2)

Based on the applied search strings and inclusion criteria, the reviewed studies primarily focused on emotional expressions, sentiment, and affective valence. They are then connected to psychosocial needs or resources of the population based on the context of the expression and the linguistic analysis. The findings were categorized following the qualitative content analysis approach outlined above via a consensus discussion of this study’s team. In total, seven psychosocial content categories were identified across the included studies: sentiment, emotions, behavior, coping strategies or resources, needs, opinions, and other psychosocial factors.

The findings indicate that (1) manual data classification and analysis tended to focus primarily on emotions; (2) sentiment was most commonly assessed using scales (eg, from −1 to +1) or categorized into positive, neutral, and negative; and (3) emotions were generally classified using nominal categories or variations of basic emotion models.

The review reveals that in the majority of studies (154/179, 86%), the deductive foundation of the chosen classification system was not explicitly stated. Among the remaining studies, commonly cited frameworks include those by Ekman [167], Plutchik [166], and Skinner [168], with Plutchik’s wheel of emotions gaining more attention in the reviewed literature since 2021. This points to a lack of clarity regarding the theoretical foundation, which makes it difficult to compare findings across studies and connect them to underlying concepts.

Most studies focusing on emotions applied a combination of several categories, such as primary and secondary emotions, anger, fear, sadness, disgust, anticipation, joy, trust, surprise, concern, terror, calmness, discomfort, anxiety, and relief. In contrast, sentiment classification was predominantly conducted using either a numerical scale (eg, −1 to +1 to describe negative or positive valence) or a three-category system (negative, neutral, or positive). Studies that compared multiple CaDs or analyzed multiple languages also aimed to consider cultural differences in expressing emotions, for instance, as coded in predefined lexica. However, these aspects were rarely co-designed or discussed with representatives from the public to ensure the validity of these approaches.

A substantial proportion of studies relied on sentiment polarity (positive, negative, neutral, or valence scales), rather than discrete emotions. The most frequently examined individual emotions were fear or anxiety, anger, and sadness, followed by joy or happiness. Disgust and surprise appeared moderately frequently, while emotions such as trust, anticipation, shame, and confusion were only sporadically considered. Methodologically, the following differences are apparent: lexicon-based approaches focused primarily on emotional polarity and emotions such as fear, anger, sadness, joy, and disgust. ML studies more frequently expanded emotion analysis to include results on, for example, anxiety, depression, stress, needs, support, and behavioral responses. Combined approaches integrated both sentiment and data-based classes, while manual coding schemes emphasized contextualized affective expressions such as grief, compassion, protest, humor, and coping strategies. The types of emotions also varied depending on the category of crises and disasters. Data from natural disasters were predominantly classified in terms of fear, anxiety, sadness, and concern. In biological crises such as epidemics and pandemics, the focus was more on psychological aspects such as fear, depression, stress, loneliness, and uncertainty. In contrast, the categories of anger, fear, grief, and blame dominated in man-made events.

Moreover, the current state of research appears to be strongly deficit-oriented. Although the search criteria of this scoping review included terms such as “health” and “well-being,” which could imply both resource- and deficit-focused approaches, the majority of studies emphasized negative sentiments and looked for indicators of negative emotions, that is, anger, fear, sadness, and stress. While this informs CaD management by pointing to potential needs, it overlooks positive emotions and psychological states. For example, offering support might be connected to galvanizing spontaneous volunteering that is promising in CaD management [169].

#### Practical Implications for Crisis and Disaster Management (RQ3)

In addition to the approach and scope of analyzing SoMe data, we were also interested in the practical implications and recommendations for decision-makers interested in implementing these techniques. Via qualitative content analysis and consensus discussions, the following implications were identified: gaining insight into population characteristics, disaster response, and situated cognitions and emotions; informing crisis communication practice; and creating learning opportunities for professionals and the public.

### Gaining Insight on Public Disaster Response

SoMe data provide real-time insights into population movements, experiences, and needs, which can improve situational assessments and awareness for evacuation planning, transportation logistics, and information dissemination during CaDs [54,85,109,149,151]. For example, connecting geolocation with a content analysis of expressed emotions and needs can help to illustrate specific needs of the local population and provide accurate support [170]. In addition, analyzing SoMe posts by language or other sociodemographic markers can point to differential processing of disaster-related information and identify opportunities for further clarification or repeated communication (eg, if original messages were not fully understood) [54,67]. Subsequent tailored communication can help to promote adaptive behavior and reduce the impact of crises [58,171]. This can include addressing mental health needs (eg, via online workshops, mental health support groups, or online therapy sessions) and aligning aid with real-time needs, such as on-site psychosocial support [51,92,139]. Following measures and interventions, monitoring SoMe response can also serve as a learning to better understand what works for whom [109,123].

Moreover, SoMe reflects the dynamic nature of public sentiment and emotional responses during crises, including their trajectories, trends, and drivers [88,91,108,172]. A close monitoring of SoMe communication over time contextualizes changes in public opinion (eg, following public statements, governmental measures, or escalating disasters), and highlights emerging concerns and actions (eg, spontaneous volunteers) [61,121]. Thus, it helps decision-makers to coordinate activities [131], connect to local resources [173], and address emerging issues [111,117,174] as well as prepare them for future crises (ie, by building anticipation and preparedness) [80,100,125,154,170].

### Informing Crisis Communication Practice

The literature points to several suggestions for improving current practice: first, establishing trust and being accessible to the public is essential [154]. Timely, transparent announcements of verified information and open government platforms can foster public trust and offer direct support during CaDs [58,66,78,102,121,127,144]. Being transparent and open regarding uncertainty and providing regular updates is perceived as trustworthy [133]. By analyzing SoMe communication, it is also possible to detect rumors and misinformation and counter them via official communication early on [67,69,80]. Second, decision-makers can build trust and capacity for crisis communication in the preparation and before a disaster strikes by connecting with relevant communities and channels (eg, local sports clubs, relief organizations, or SoMe influencers) that might be of importance in disaster management efforts [122]. It is also recommended to use verified accounts by official organizations and to prioritize dissemination through news media [54,85,121,139]. This should also include “traditional media” (eg, radio or newspapers) to reach audiences that are not represented in SoMe spaces [85,175]. Third, crisis communication should be mindful of balancing disaster-related information and reports with galvanizing and resource-building communication. For instance, a communication that frames a situation as a challenge rather than a threat may help reduce public anger and build optimism and resilience [133], while acknowledging public fears and showing empathy can be more effective than purely factual messaging [88]. Therefore, addressing emotions is important, and particularly positive states (eg, hope or optimism) should be reiterated to boost collective efficacy [122,175]. This can be achieved by pointing to positive local actions, such as volunteers, highlighting experiences of resilience, and offering opportunities for action (eg, hotlines, support groups, or volunteering) to avoid helplessness and lack of control [65]. Nevertheless, these strategies require skills in adequately expressing needs and emotions, recognizing them, and initiating appropriate action.

### Learning Opportunities for Professionals and the Public

Consequently, disaster management training should explicitly integrate SoMe use and include educational measures to guide the public’s information-sharing behavior in crisis contexts [47,58,82,133,138,171]. For professionals, this requires training in working with SoMe, connecting to specific resources (eg, social listening tools, monitoring centers, or virtual operation support teams) and including dedicated professionals in crisis management teams (eg, specialists for social listening) [141,176]. Communication efforts should also continue beyond the acute phase, as threats may persist and public vigilance remains necessary [141]. Therefore, professionals need to know how to continuously work with SoMe in these contexts and maintain relationships. For the public, it is important to be able to express needs, use resources, and manage SoMe communication during a crisis. This requires critical media literacy, SoMe-related skills, and empathy and emotion regulation (ie, recognizing, understanding, and communicating emotions in the self and in others) [88,133,141]. For example, experienced SoMe managers in volunteer organizations can moderate online discussions, guide toward verified and trustworthy information, and coordinate online efforts (eg, donation campaigns) [177]. However, additional education and practice are needed to build these skills and be confident enough to implement them in times of crisis. Therefore, decision-makers should integrate relevant stakeholders into training and scenarios, and agencies should allocate sufficient resources (time and finances) to training their staff and cooperating with public stakeholders. Finally, it is important to have an open mind to lifelong learning to be able to discover, understand, and integrate innovation in the crisis communication space (eg, personalized AI communication tools, augmented or virtual reality simulations, and their impact on communication habits, processes, and consequences) [122].

Further learning opportunities refer to the gaps in knowledge and practice that remain. As positive emotions were not a focus of most studies, there is insufficient evidence on how SoMe might build public resilience and community capacity during CaDs, although this was underlined as a key factor for successful disaster management. Additionally, most studies conducted retrospective analyses of reactions to acute CaD events; it is unclear how SoMe use changes over time and with it the expression of psychosocial needs. Many of the aforementioned pathways are assumed based on the analysis, but it is difficult to test them in real-world scenarios due to ethical (eg, comparing more and presumably less successful communication strategies), conceptual (eg, it is challenging to control for all threats to validity), and structural (eg, lack of resources to conduct research on ongoing CaD management processes) constraints. Yet, overall, the potential of social media analysis (SMA) is consistently emphasized. It supports real-time situational awareness and accelerates decision-making and resource allocation in time-sensitive scenarios [59,97,137,140]. SMA also enables the early detection of emerging problems, identification of need patterns, and mental health risks [175,178], which in turn aid the design of long-term recovery and resilience strategies [103].

## Discussion

### Principal Findings

This scoping review provides an overview of the current research landscape regarding the analysis of SoMe data during CaDs to obtain information about mental health status, affective states, and potential needs in the general population. It identified 179 studies that report on SoMe analysis during CaDs and summarizes methodological approaches (RQ1), key topics (RQ2), and recommendations for decision-makers and practitioners (RQ3) regarding CaD management. Overall, most studies examined natural disasters, used lexicon-based approaches to analyze SoMe data, but did not explicitly state a theoretical foundation for the analysis of psychosocial dimensions. The studies were retrospective and focused on the acute CaD phase; thus, implications are limited, and more research is needed on capturing long-term changes in SoMe use, mental health status, needs, and resources in the population following CaDs. Moreover, no study tested a model during an active CaDs with sequential data availability, raising concerns about real-world applicability.

Regarding the operationalization of mental health, most studies focused on primary or secondary emotions [166,167], oftentimes anxiety, fear, stress, or sadness derived from relevant lexica. In many cases, the sentiment was assessed using scales (eg, from −1 to +1) or categorized into positive, neutral, and negative; and in some studies, the intensity of expressed sentiment or emotions was captured. While many studies also described needs and coping mechanisms in their primary data, the analytical approach was less clear. Assessment methods varied between studies; they were rarely linked to established measures or models and thus rarely validated (eg, psychometrically tested scales and conceptual models of depression or anxiety) [128]. Positive emotions were investigated less frequently.

Finally, recommendations for practitioners and policymakers were addressed in the majority of the included studies. They ranged from structural recommendations (eg, provision of financial or educational resources) to process-oriented recommendations (eg, regarding crisis communication framing) and outcome-oriented recommendations (eg, including collective capacity as relevant outcomes). However, they were rarely tested or implemented in the included studies, challenging their potential for real-world implementation.

### Contextualization of the Findings

A major result of the review is the dominance of natural disasters in the literature, accounting for 76% (203/267) of crisis types analyzed. In contrast, Huang et al [179] identified only 35% of SoMe-detected CaDs as natural and 42% as anthropogenic. Similarly, findings diverge from Zhang et al [37], particularly regarding meteorological events, which represent 21% (56/267) of cases in this review. We assume that an increasing interest in climate change and climate-related crises and disasters in recent years, as well as experiences of global disasters (eg, the COVID-19 pandemic), might have led to an increasing number of publications addressing natural disasters and their impact.

Geographic distribution also reveals a research imbalance. While Tin et al [180] reported that 39% of global natural disasters occur in Asia, 24% in the Americas, and 21% in Africa, only 1 included study in this review addressed a CaD in Africa. Although most reviewed studies focus on Asia and the Americas—possibly reflecting CaD frequency—Africa and the most severe CaDs (by death toll) remain underrepresented. Only one of the ten deadliest disasters, the Haiti earthquake [151], was examined in this dataset. Additionally, only 9% (16/179) of the sample considered both natural disasters and public health aspects, although this marks an increase from the 2% reported by Suhaimin et al [35]. For the German context, only 5 studies were identified, only one of which addressed storms, floods, or extreme temperatures—despite these accounting for a substantial proportion of CaDs in Germany [180]. This highlights a gap in the analysis of German SoMe data about prevalent CaD types, suggesting a need for localized research to test the transferability of international findings.

The review also shows considerable heterogeneity across disciplines, ranging from technical and algorithmic studies to investigations of collective behavior and psychosocial impact [9,81,136]. Several key areas were identified, such as sentiment detection, emotional response, needs, and coping mechanisms. The detailed description of the most commonly used lexicon-based and ML models (Table 1 and Multimedia Appendix 3) is helpful for extended research on emotion and sentiment detection and classification in SoMe posts and might help to differentiate more complex emotions, such as guilt or shame, in future analyses. It is important groundwork for defining categories and parameters to test large language models and train ML models for future studies. In this sense, our review reiterates findings from previous work, such as Zhang et al [37]. However, as most studies used lexicon-based tools and focused on sentiments and emotions, further validation is needed regarding needs and coping mechanisms. As emotions are contextualized and can have different functions and lead to different outcomes (eg, a SoMe-based complaint about government policies can express factual criticism, individual anger or fear, and gather social support or mistrust) [10-16], it is paramount to better understand and consider the context in which mental health-related information is shared on SoMe. Some studies suggest an iterative, stepwise approach where computer science (eg, recognizing and describing emotions) and psychological and social science (eg, interpreting emotions and recommended actions) work together to optimize detection, interpretation, and use of SoMe analytics in CaDs. However, this requires considerable resources and expertise to develop, prepare, implement, and maintain the process. Future research could examine how and when such collaboration is fruitful, what the conditions and requirements are, and provide guidance on their real-world implementation.

Currently, this perspective is further limited by the retrospective nature of most studies. Building on large corpora of SoMe posts, the researchers were able to analyze and reconstruct communication patterns on SoMe [12,52,56,135]. However, it is unclear how these approaches can be applied in real-world contexts, with high stress, time pressure, and scarce resources (eg, regarding SoMe communication between authorities and the public). Therefore, more research is needed, using experimental designs and living laboratories, to implement and evaluate these strategies and approaches under ecologically valid conditions. Moreover, current research appears to be strongly deficit-oriented, that is, most studies emphasized negative sentiments and looked for indicators of negative emotions, such as anger, fear, sadness, and stress (Multimedia Appendix 3), although search terms such as “well-being” were also included. While this informs CaD management by pointing to potential needs, it overlooks positive emotions and psychological states that can also arise during CaDs. For example, spontaneous volunteering is promising in CaD management [169] and is supported by the wish to contribute and be efficacious in the face of a seemingly uncontrollable threat. Accordingly, recommendations for practitioners also state that crisis communication should incorporate good practice examples (eg, of spontaneous volunteers) and positive narratives (eg, of resilient individuals) to foster hope, optimism, and build resilience. Interestingly, this aspect was rarely reflected in the identified studies, pointing to a clear gap. More research is needed to examine indicators of positive emotions; analyze their trajectories; and test interventions to build, boost, and maintain them across time.

Despite promising technical results, for instance, regarding lexicon-based tools and ML models, the review also points to gaps regarding the integration of user perspectives. The development of algorithms for ML-based approaches and the selection of lexicon-based tools is often performed by a research team but rarely informed by end users, for instance, policymakers or members of the public who are supposed to be represented in the SoMe communication and benefit from the analysis and the findings. This also relates to previous research on motives of SoMe use in CaDs and the role of practitioners in this context [47,58,82,133,138,171]. As pointed out in the implications for practice section Practical Implications for Crisis and Disaster Management (RQ3), ongoing training and integration into disaster management routines are paramount to ensure implementation fidelity, usefulness, and sustainable implementation. If expectations and requirements of decision-makers, practitioners, and public stakeholders are not accounted for, there is a chance that the developed tools and techniques will not be implemented or will be wrongfully used (eg, findings could be misinterpreted or aspects such as positive emotions could be overlooked if they are not fully represented). To ensure validity, reduce the possibility of harm and unintended side-effects, and improve real-world impact, it is therefore recommended to include participatory action research and end user perspectives more strongly in this line of research.

### Limitations

This review is subject to several limitations. First, while the review followed an established JBI scoping review methodology, adhered to the PRISMA-S [41] and PRISMA-ScR protocol [31], and combined qualitative and quantitative methods, the heterogeneity of the included studies makes direct comparisons challenging. Second, despite covering literature up to 2025, there may be a publication lag or underrepresentation of emerging or non-English studies (searches were limited to English and German, thus excluding other languages). Third, the review focused on publicly available peer-reviewed literature and three key databases (PubMed, Web of Science, and Scopus), potentially omitting valuable insights from gray literature, literature unique to other databases, or unpublished projects, especially those led by governments or nongovernmental organizations. Fourth, although the findings highlight the potential of SoMe for psychosocial insight, most tools and methods have not been evaluated in real-time or operational settings. Finally, the geographic bias toward studies from Asia and the Americas, and the underrepresentation of CaDs in Africa, limit generalizability.

### Conclusions

This scoping review highlights both the potential and the current limitations of using SoMe analytics to derive psychosocial insights in crisis and disaster contexts to inform disaster management. Although various technical solutions exist and perform well in retrospective analyses, they often are unclear on theoretical grounding, user-centered design, and operational testing. Hence, more real-time data analysis and testing under realistic conditions (eg, via living laboratories or highly immersive simulations) are needed. Nevertheless, the research presents several models and approaches that can be beneficial in identifying distinct sentiments and emotions in large datasets, which could serve as indicators of specific mental health states or point to specific needs. Regarding the content of the analysis, the focus on negative emotions or states is a challenge for disaster management, as it neglects the populations’ own coping resources. Research on resilience and positive coping shows that several efforts, such as volunteering, sharing emotions, and forming new social bonds and identities (eg, as survivors of a specific disaster or condition), can be beneficial to mental health and build social capital to tackle future challenges. While these trends are also reflected in the literature on coping, mental health, and SoMe in CaDs at large, it seems that they are not fully connected to the area of SoMe analytics yet. Thus, this is a task for future research. Finally, the findings underscore the need for future work to close the gap between available technologies and actual user requirements, especially among decision-makers. To enhance the practical applicability of SMA, future applications should be transparent, interpretable, and embedded in strategies that support evidence-based communication strategies, adequate emotional regulation, and resource planning during CaDs. This should also be connected to an equity-informed approach that considers regional differences, regarding knowledge, practices, and capacities (eg, in regions that are highly affected by disasters but lack education and resources for disaster management), in building global resilience.

## Supplementary material

10.2196/79762 Multimedia Appendix 1 Overview of compared reviews and excluded studies.

10.2196/79762 Multimedia Appendix 2 Search strategy.

10.2196/79762 Multimedia Appendix 3 Overview of included studies and analytical categories.

10.2196/79762 Checklist 1 PRISMA-ScR checklist.

10.2196/79762 Checklist 2 PRISMA-S checklist.

## Abbreviations

CaD: crisis and disaster situation
EHEC: enterohemorrhagic Escherichia coli
EM-DAT: Emergency Events Database
JBI: Joanna Briggs Institute
MeSH: Medical Subject Headings
ML: machine learning
PRISMA-S: Preferred Reporting Items for Systematic Reviews and Meta-Analyses literature search extension
PRISMA-ScR: Preferred Reporting Items for Systematic Reviews and Meta-Analyses extension for Scoping Reviews
SMA: social media analysis
SoMe: social media
VADER: Valence Aware Dictionary and Sentiment Reasoner
