# Supplementary material for: Analyzing Social Media to Infer Mental Health Status and Affective States for Crisis and Disaster Management: Scoping Review
Source: J Med Internet Res. 2026 Jul 20;28:e79762. doi: 10.2196/79762 (PMC13384355; doi:10.2196/79762)
Supplement: Multimedia Appendix 1 — Overview of compared reviews and excluded studies. [file jmir-v28-e79762-s001.docx]

**Overview of compared reviews and excluded studies**

**S1.1. Overview of compared reviews**

The reviews were identified using the search string, see Multimedia Appendix 3, and the snowball principle, analogous to the scoping review procedure. The following table lists the reviews that were assessed as similar to the present review on the basis of the title and abstract, but could be differentiated on the basis of the full text. The reasons for the differentiation are also listed.

A table showing the comparison procedure and the inclusion and exclusion criteria of the compared reviews can be provided on request to the author.

| **Title** | **First Author (Year)** | **Differentiation based on full-text screening** |
| --- | --- | --- |
| Social Media Use in Emergency Response to Natural Disasters: A Systematic Review With a Public Health Perspective | Muniz-Rodriguez [28](2020)  (44 studies) | While the comparative study is limited to a narrower period from 2015 to 2018 and natural events, the study presented here extends the period under consideration and does not exclude any types of disasters in order to capture current developments in the use of social media. Furthermore, the analysis presented here goes beyond the focus on warning dissemination and needs identification and considers a broader range of applications and impacts of social media in disaster management. Another key difference is that our study explicitly includes psychological and psychosocial factors, which are not specifically addressed in the comparative study, providing a more comprehensive picture of the multidimensional role of social media in disaster situations. |
| Methods and Applications of Social Media Monitoring of Mental Health During Disasters: Scoping Review | Teague [29] (2022)  (47 studies) | Firstly, the analysis presented here extends the investigation period by almost two years, which enables a more up-to-date view of the research field. Furthermore, the methodology regarding the application of the search string in IEEE Xplore is unclear. While the comparative study is limited to specific analysis methods such as machine learning and data mining as well as the identification of psychological disorders, the study presented here broadens the focus and also includes studies that do not exclusively use these methods or only refer to psychological conditions, which covers a broader spectrum of research approaches. In addition, the study presented here explicitly considers the broader crisis context, looks at developments over time and analyzes how the information gained can be used practically for crisis management. |
| A Systematic Review of Techniques Employed for Determining Mental Health Using Social Media in Psychological Surveillance During Disasters | Karmegam [30] (2019)  (18 studies) | While this study is limited to the period from 2009 to 2018, the analysis presented here extends the study period considerably, which leads to a much more up-to-date and comprehensive overview given the 4335 new publications in PubMed alone since 2019. In addition, the study presented here uses an extended search string, which enables a broader coverage of relevant literature. In contrast to the comparative study, the study presented here places particular emphasis on analyzing temporal developments in the research field and explicitly considers how the information obtained can be used practically for crisis management, thus creating a stronger practical relevance. Overall, however, this study provides a good basis for comparison with the study presented here. |
| Are Emotion-Expressing Messages More Shared on Social Media? A Meta-Analytic Review | Chen [31] (2022)  (19 studies) | This study focuses on the influence of emotionally expressive messages on the population. The study presented here concentrates on the derivation and interpretation of emotionally expressive messages from social media to gain information in a crisis context. |
| Collective behaviour, social media, and disasters: A systematic literature review | Eismann [32] (2016)  (68 studies) | While collective behavior is the focus of this article, the scoping review presented here focuses on the role of social media in relation to psychological factors and support for those affected. In addition, this paper looks at different types of crises, sentiment, emotion, behavior and opinion analyses without limiting itself exclusively to behavioral phenomena. |
| Social media sentiment analysis and opinion mining in public security: Taxonomy, trend analysis, issues and future directions | Suhaimin [33] (2023)  (200 studies) |  |
| An overview of sentiment analysis in social media and its applications in disaster relief | Beigi [36] (2016)  (not named) |  |
| The role of social media in public health crises caused by infectious disease: a scoping review | Terry [37] (2023)  (338 studies) | While the article examines social media primarily in the context of public health crises caused by infectious diseases and treats mental health only as a secondary topic, the work presented here focuses on psychosocial factors. In addition, different types of crises and disasters are included to provide a broader understanding of the role of social media in these contexts. |
| Social media for intelligent public information and warning in disasters: An interdisciplinary review | Zhang [35] (2019)  (304 studies) | The difference to this article lies in the different thematic focus. While the compared article examines communication phenomena in social media during disasters as well as data analysis techniques for the collection and dissemination of critical information, this paper focuses on the derivation of psychosocial factors. Sentiment and psychosocial information, which play no role in the other article, are therefore central elements of the study presented here. |

**References:**

28. Muniz-Rodriguez K, Ofori SK, Bayliss LC, et al. Social media use in emergency response to natural disasters: a systematic review with a public health perspeMedia Use in Emergency Response to Natural Disasters: A Systematic Review With a Public Health Perspective. Disaster Med Public Health Prep. Feb 2020;14(1):139-149. [doi: 10.1017/dmp.2020.3] [Medline: 32148219]

29. Teague SJ, Shatte ABR, Weller E, Fuller-Tyszkiewicz M, Hutchinson DM. Methods and applications of social media monitoring of mental health during disasters: scoping re Applications of Social Media Monitoring of Mental Health During Disasters: Scoping Review. JMIR Ment Health. Feb 28, 2022;9(2):e33058. [doi: 10.2196/33058] [Medline: 35225815]

30. Karmegam D, Ramamoorthy T, Mappillairajan B. A systematic review of techniques employed for determining mental health using social media in psychological surveillance during disSystematic Review of Techniques Employed for Determining Mental Health Using Social Media in Psychological Surveillance During Disasters. Disaster Med Public Health Prep. Apr 2020;14(2):265-272. [doi: 10.1017/dmp.2019.40] [Medline: 31272518]31. Chen J, Yan Y, Leach J. Are emotion-expressing messages more shared on social media? A meta-analytic review. Rev Commun Res. 2022;10:59-79. [doi: 10.12840/ISSN.2255-4165.034]

32. Eismann K, Posegga O, Fischbach K. Collective behaviour, social media, and disasters: a systematic literature review. Vol 104. Jun 15, 2016:1-21. URL: <http://aisel.aisnet.org/ecis2016_rp/104> [Accessed 2026-05-29]

33. Suhaimin MSM, Hijazi MHA, Moung EG, Nohuddin PNE, Chua S, Coenen F. Social media sentiment analysis and opinion mining in public security: taxonomy, trend analysis, issues and future directions. J King Saud Univ - Comput Inf Sci. Oct 2023;35(9):101776. [doi: 10.1016/j.jksuci.2023.101776] [Medline: 39071961]

36. Beigi G, Hu X, Maciejewski R, Liu H. An overview of sentiment analysis in social media and its applications in disaster relief. In: Studies in Computational Intelligence. Springer, Cham; 2016:313-340. [doi: 10.1007/978-3-319-30319-2_13]

37. Terry K, Yang F, Yao Q, Liu C. The role of social media in public health crises caused by infectious disease: a scoping review. BMJ Glob Health. Dec 28, 2023;8(12):e013515. [doi: 10.1136/bmjgh-2023-013515] [Medline: 38154810]

35. Zhang C, Fan C, Yao W, Hu X, Mostafavi A. Social media for intelligent public information and warning in disasters: aAn interdisciplinary review. Int J Inf Manage. Dec 2019;49:190-207. [doi: 10.1016/j.ijinfomgt.2019.04.004]

**S1.2. Overview of excluded studies based on full-text**

| **First author (Year)** | **title** | **exclusion based on full-text screening** |
| --- | --- | --- |
| Jin  (2012) | Toward a publics-driven, emotion-based conceptualization in crisis communication: Unearthing dominant emotions in multi-staged testing of the integrated crisis mapping (ICM) model | no social media, only news as data |
| Black  (2015) | Do social media have a place in public health emergency response? | No access |
| Miura  (2015) | Expression of negative emotional responses to the 2011 Great East Japan Earthquake: Analysis of big data from social media | Chinese language |
| Palen (2016) | SOCIAL MEDIA RESEARCH. Crisis informatics--New data for extraordinary times |  |
| Bala  (2017) | Sentiment trends on natural disasters using location based twitter opinion mining | disaster not named, just mentioned that it would be disaster context, many spelling mistakes |
| Coskun  (2018) | #europehappinessmap: A Framework for Multi-Lingual Sentiment Analysis via Social Media Big Data (A Twitter Case Study) | No focus on disaster situation |
| Hassan  (2019) | Sentiment analysis from images of natural disasters | disaster not named |
| Kaufhold  (2019) | SentiNet: Twitter-basierter Ansatz zur kombinierten Netzwerk- und Stimmungsanalyse in Katastrophenlagen | No focus on specific disaster situation |
| Prabhakar  (2019) | Climate change and twitter – An empirical analysis of environmental awareness and engagement | No access |
| Sano (2019) | Identifying long-term periodic cycles and memories of collective emotion in online social media | No crisis |
| Sadiq (2020) | Human Sentiment and Activity Recognition in Disaster Situations Using Social Media Images Based on Deep Learning |  |
| Devi  (2021) | Location Based Twitter Emotion Classification for Disaster Management | obwohl Ansatz Entwicklung einer simplen Methode für DM -> kein CaD benannt |
| Lee (2021) | Using Twitter to Understand the Effects of the Cameroon Anglophone Crisis on Social Determinants of Health |  |
| Hassan (2022) | Visual Sentiment Analysis from Disaster Images in Social Media |  |
| Cao  (2023) | Analysis of the Emotional Characteristics of People in Earthquake-stricken Areas Based on Social Media Data Mining | No access |
| Kanungo  (2023) | Hybrid Deep Neural Network G-LSTM for Sentiment Analysis on Twitter: A Novel Approach to Disaster Management | disaster nicht eindeutig definierbar -> lediglich Benennung, dass ein Teil vom Datensatz disaster Bezug hat |
| Minocha  (2023) | A Novel Sentimental Analysis for Response to Natural Disaster on Twitter Data | No access |
