# Supplementary material for: Analyzing Social Media to Infer Mental Health Status and Affective States for Crisis and Disaster Management: Scoping Review
Source: J Med Internet Res. 2026 Jul 20;28:e79762. doi: 10.2196/79762 (PMC13384355; doi:10.2196/79762)
Supplement: Multimedia Appendix 2 — Search strategy. [file jmir-v28-e79762-s002.docx]

**Search Strategy**

**S1.1 Literature Search Strategy**

We used two different search strings. The first search string aimed to identify reviews based on the same extended search string to focus the same topics. The second search string aimed to identify studies that focus on the connection of the following topics:

- Social media,
- crisis and disaster situation and
- mental health of the population.

Synonyms were added on the basis of an unstructured literature search. Specific social media platforms (e.g., Twitter, WhatsApp, Instagram, TikTok) were included as search terms to capture studies that explicitly refer to these services. As many platform names are not consistently represented in controlled vocabularies such as MeSH, these terms were primarily used as free-text search terms.

The search strategy combined field-restricted keyword searches with controlled vocabulary terms where available. Specific social media platforms (e.g., Twitter, WhatsApp, Instagram, TikTok) were included as keywords to capture studies explicitly referring to these services, as such platforms are not consistently represented in controlled vocabularies such as MeSH. Consequently, these terms primarily functioned as title-based keyword searches.

Exclusion criteria were added on the basis of iterative adaptation as part of the database search

- Exclusion of marketing via “brand*”, “consumer*” and “buy*”
- Exclusion of financial foci via “financial*”
- Exclusion of medical foci via “medication*”

The search strings were implemented in English and in German

The full search strings, including the synonyms in each database, can be found below.

First search was done in July 2023, updated on the 08/03/2024 and finally, on the 27/11/2025.

1. Web of Science

(TI=("social media*") OR AK=("social media*") OR TI=("social network*") OR AK=("social network*") OR TI=("user generated data") OR AK=("user generated data") OR TI=(twitter) OR AK=(twitter) OR TI=(linkedin) OR AK=(linkedin) OR TI=(whatsapp) OR AK=(whatsapp) OR TI=(xing) OR AK=(xing) OR TI=(pinterest) OR AK=(pinterest) OR TI=(snapchat) OR AK=(snapchat) OR TI=(instagram) OR AK=(instagram) OR TI=(telegram) OR AK=(telegram) OR TI=(reddit) OR AK=(reddit) OR TI=(facebook) OR AK=(facebook) OR TI=(youtube) OR AK=(youtube) OR TI=(tiktok) OR AK=(tiktok) OR TI=(weibo) OR AK=(weibo)) AND (TI=(emergenc*) OR AK=(emergenc*) OR TI=("extreme event*") OR AK=("extreme event*") OR TI=(crisis) OR AK=(crisis) OR TI=(crises) OR AK=(crises) OR TI=(disaster*) OR AK=(disaster*) OR TI=(catastrophe*) OR AK=(catastrophe*) OR TI=(traged*) OR AK=(traged*) OR TI=(hazard*) OR AK=(hazard*)) AND (TI=(psycho*) OR AK=(psycho*) OR TI=("mental health") OR AK=("mental health") OR TI=(behav*) OR AK=(behav*) OR TI=(wellbeing) OR AK=(wellbeing) OR TI=(well-being) OR AK=(well-being) OR TI=(mood) OR AK=(mood) OR TI=(emotion) OR AK=(emotion) OR TI=(sentiment) OR AK=(sentiment) OR TI=(stress*) OR AK=(stress*) OR TI=(distress*) OR AK=(distress*)) NOT ALL=(brand*) NOT ALL=(consumer*) NOT ALL=(buy*) NOT ALL=(medication*) NOT ALL=(marketing*) NOT ALL=(financial*)

+ AND TI=review*

(TI=("soziale medien*") OR AK=("soziale medien*") OR TI=("soziale netzwerk*") OR AK=("soziale netzwerk*") OR TI=("nutzergenerierte daten") OR AK=("nutzergenerierte daten") OR TI=(twitter) OR AK=(twitter) OR TI=(linkedin) OR AK=(linkedin) OR TI=(whatsapp) OR AK=(whatsapp) OR TI=(xing) OR AK=(xing) OR TI=(pinterest) OR AK=(pinterest) OR TI=(snapchat) OR AK=(snapchat) OR TI=(instagram) OR AK=(instagram) OR TI=(telegram) OR AK=(telegram) OR TI=(reddit) OR AK=(reddit) OR TI=(facebook) OR AK=(facebook) OR TI=(youtube) OR AK=(youtube) OR TI=(tiktok) OR AK=(tiktok) OR TI=(weibo) OR AK=(weibo)) AND (TI=(notfall*) OR AK=(notfall*) OR TI=("Extremereignis*") OR AK=("Extremereignis*") OR TI=(krise*) OR AK=(krise*) OR TI=(disaster*) OR AK=(disaster*) OR TI=(katastrophe*) OR AK=(katastrophe*) OR TI=(tragödie*) OR AK=(tragödie*) OR TI=(gefahr*) OR AK=(gefahr*)) AND (TI=(psych*) OR AK=(psych*) OR TI=("mental health") OR AK=("mental health") OR TI=(verhalten*) OR AK=(verhalten*) OR TI=(wohlbefinden) OR AK=(wohlbefinden) OR TI=(wellbeing) OR AK=(wellbeing) OR TI=(stimmung) OR AK=(stimmung) OR TI=(emotion) OR AK=(emotion) OR TI=(gefühl*) OR AK=(gefühl*) OR TI=(sentiment) OR AK=(sentiment) OR TI=(stress*) OR AK=(stress*) OR TI=(distress*) OR AK=(distress*)) NOT ALL=(marke*) NOT ALL=(konsument*) NOT ALL=(kauf*) NOT ALL=(medika*) NOT ALL=(marketing*) NOT ALL=(finanz*)

1. Scopus

(TITLE("social media*") OR AUTHKEY("social media*") OR TITLE("social network*") OR AUTHKEY("social network*") OR TITLE("user generated data") OR AUTHKEY("user generated data") OR TITLE(twitter) OR AUTHKEY(twitter) OR TITLE(linkedin) OR AUTHKEY(linkedin) OR TITLE(whatsapp) OR AUTHKEY(whatsapp) OR TITLE(xing) OR AUTHKEY(xing) OR TITLE(pinterest) OR AUTHKEY(pinterest) OR TITLE(snapchat) OR AUTHKEY(snapchat) OR TITLE(instagram) OR AUTHKEY(instagram) OR TITLE(telegram) OR AUTHKEY(telegram) OR TITLE(reddit) OR AUTHKEY(reddit) OR TITLE(facebook) OR AUTHKEY(facebook) OR TITLE(youtube) OR AUTHKEY(youtube) OR TITLE(tiktok) OR AUTHKEY(tiktok) OR TITLE(weibo) OR AUTHKEY(weibo)) AND (TITLE(emergenc*) OR AUTHKEY(emergenc*) OR TITLE("extreme event*") OR AUTHKEY("extreme event*") OR TITLE(crisis) OR AUTHKEY(crisis) OR TITLE(crises) OR AUTHKEY(crises) OR TITLE(disaster*) OR AUTHKEY(disaster*) OR TITLE(catastrophe*) OR AUTHKEY(catastrophe*) OR TITLE(traged*) OR AUTHKEY(traged*) OR TITLE(hazard*) OR AUTHKEY(hazard*)) AND (TITLE(psycho*) OR AUTHKEY(psycho*) OR TITLE("mental health") OR AUTHKEY("mental health") OR TITLE(behav*) OR AUTHKEY(behav*) OR TITLE(wellbeing) OR AUTHKEY(wellbeing) OR TITLE(well-being) OR AUTHKEY(well-being) OR TITLE(mood) OR AUTHKEY(mood) OR TITLE(emotion) OR AUTHKEY(emotion) OR TITLE(sentiment) OR AUTHKEY(sentiment) OR TITLE(stress*) OR AUTHKEY(stress*) OR TITLE(distress*) OR AUTHKEY(distress*)) AND NOT ALL(brand*) AND NOT ALL(consumer*) AND NOT ALL(buy*) AND NOT ALL(medication*) AND NOT ALL(marketing*) AND NOT ALL(financial*)

+ AND TITLE(review)

(TITLE("soziale medien*") OR AUTHKEY("soziale medien*") OR TITLE("soziale netzwerk*") OR AUTHKEY("soziale netzwerk*") OR TITLE("nutzergenerierte daten") OR AUTHKEY("nutzergenerierte daten") OR TITLE(twitter) OR AUTHKEY(twitter) OR TITLE(linkedin) OR AUTHKEY(linkedin) OR TITLE(whatsapp) OR AUTHKEY(whatsapp) OR TITLE(xing) OR AUTHKEY(xing) OR TITLE(pinterest) OR AUTHKEY(pinterest) OR TITLE(snapchat) OR AUTHKEY(snapchat) OR TITLE(instagram) OR AUTHKEY(instagram) OR TITLE(telegram) OR AUTHKEY(telegram) OR TITLE(reddit) OR AUTHKEY(reddit) OR TITLE(facebook) OR AUTHKEY(facebook) OR TITLE(youtube) OR AUTHKEY(youtube) OR TITLE(tiktok) OR AUTHKEY(tiktok) OR TITLE(weibo) OR AUTHKEY(weibo)) AND (TITLE(notfall*) OR AUTHKEY(notfall*) OR TITLE("Extremereignis*") OR AUTHKEY("Extremereignis*") OR TITLE(krise*) OR AUTHKEY(krise*) OR TITLE(disaster*) OR AUTHKEY(disaster*) OR TITLE(katastrophe*) OR AUTHKEY(katastrophe*) OR TITLE(tragödie*) OR AUTHKEY(tragödie*) OR TITLE(gefahr*) OR AUTHKEY(gefahr*)) AND (TITLE(psych*) OR AUTHKEY(psych*) OR TITLE("mental health") OR AUTHKEY("mental health") OR TITLE(verhalten*) OR AUTHKEY(verhalten*) OR TITLE(wohlbefinden) OR AUTHKEY(wohlbefinden) OR TITLE(wellbeing) OR AUTHKEY(wellbeing) OR TITLE(stimmung) OR AUTHKEY(stimmung) OR TITLE(emotion) OR AUTHKEY(emotion) OR TITLE(gefühl*) OR AUTHKEY(gefühl*) OR TITLE(sentiment) OR AUTHKEY(sentiment) OR TITLE(stress*) OR AUTHKEY(stress*) OR TITLE(distress*) OR AUTHKEY(distress*)) AND NOT ALL(marke*) AND NOT ALL(konsument*) AND NOT ALL(kauf*) AND NOT ALL(medika*) AND NOT ALL(marketing*) AND NOT ALL(finanz*)

1. PubMed

(("social media*"[Title]) OR ("social media*"[MeSH Terms]) OR ("social network*"[Title]) OR ("social network*"[MeSH Terms]) OR ("user generated data"[Title]) OR ("user generated data"[MeSH Terms]) OR (twitter[Title]) OR (twitter[MeSH Terms]) OR (linkedin[Title]) OR (linkedin[MeSH Terms]) OR (whatsapp[Title]) OR (whatsapp[MeSH Terms]) OR (xing[Title]) OR (xing[MeSH Terms]) OR (pinterest[Title]) OR (pinterest[MeSH Terms]) OR (snapchat[Title]) OR (snapchat[MeSH Terms]) OR (instagram[Title]) OR (instagram[MeSH Terms]) OR (telegram[Title]) OR (telegram[MeSH Terms]) OR (reddit[Title]) OR (reddit[MeSH Terms]) OR (facebook[Title]) OR (facebook[MeSH Terms]) OR (youtube[Title]) OR (youtube[MeSH Terms]) OR (tiktok[Title]) OR (tiktok[MeSH Terms]) OR (weibo[Title]) OR (weibo[MeSH Terms])) AND ((emergenc*[Title]) OR (emergenc*[MeSH Terms]) OR ("extreme event*"[Title]) OR ("extreme event*"[MeSH Terms]) OR (crisis[Title]) OR (crisis[MeSH Terms]) OR (crises[Title]) OR (crises[MeSH Terms]) OR (disaster*[Title]) OR (disaster*[MeSH Terms]) OR (catastrophe*[Title]) OR (catastrophe*[MeSH Terms]) OR (traged*[Title]) OR (traged*[MeSH Terms]) OR (hazard*[Title]) OR (hazard*[MeSH Terms])) AND ((psycho*[Title]) OR (psycho*[MeSH Terms]) OR ("mental health"[Title]) OR ("mental health"[MeSH Terms]) OR (behav*[Title]) OR (behav*[MeSH Terms]) OR (wellbeing[Title]) OR (wellbeing[MeSH Terms]) OR (well-being[Title]) OR (well-being[MeSH Terms]) OR (mood[Title]) OR (mood[MeSH Terms]) OR (emotion[Title]) OR (emotion[MeSH Terms]) OR (sentiment[Title]) OR (sentiment[MeSH Terms]) OR (stress*[Title]) OR (stress*[MeSH Terms]) OR (distress*[Title]) OR (distress*[MeSH Terms])) NOT (brand*) NOT (consumer*) NOT (buy*) NOT (medication*) NOT (marketing*) NOT (financial*)

+ AND (review*[Title])

(("soziale medien*"[Title]) OR ("soziale medien*"[MeSH Terms]) OR ("soziale netzwerk*"[Title]) OR ("soziale netzwerk*"[MeSH Terms]) OR ("nutzergenerierte daten"[Title]) OR ("nutzergenerierte daten"[MeSH Terms]) OR (twitter[Title]) OR (twitter[MeSH Terms]) OR (linkedin[Title]) OR (linkedin[MeSH Terms]) OR (whatsapp[Title]) OR (whatsapp[MeSH Terms]) OR (xing[Title]) OR (xing[MeSH Terms]) OR (pinterest[Title]) OR (pinterest[MeSH Terms]) OR (snapchat[Title]) OR (snapchat[MeSH Terms]) OR (instagram[Title]) OR (instagram[MeSH Terms]) OR (telegram[Title]) OR (telegram[MeSH Terms]) OR (reddit[Title]) OR (reddit[MeSH Terms]) OR (facebook[Title]) OR (facebook[MeSH Terms]) OR (youtube[Title]) OR (youtube[MeSH Terms]) OR (tiktok[Title]) OR (tiktok[MeSH Terms]) OR (weibo[Title]) OR (weibo[MeSH Terms])) AND ((notfall*[Title]) OR (notfall*[MeSH Terms]) OR ("extremereignis*"[Title]) OR ("extremereignis*"[MeSH Terms]) OR (krise[Title]) OR (krise[MeSH Terms]) OR (disaster*[Title]) OR (disaster*[MeSH Terms]) OR (katastrophe*[Title]) OR (katastrophe*[MeSH Terms]) OR (tragödie*[Title]) OR (tragödie*[MeSH Terms]) OR (gefahr*[Title]) OR (gefahr*[MeSH Terms])) AND ((psych*[Title]) OR (psych*[MeSH Terms]) OR ("mental health"[Title]) OR ("mental health"[MeSH Terms]) OR (verhalten*[Title]) OR (verhalten*[MeSH Terms]) OR (wellbeing[Title]) OR (wellbeing[MeSH Terms]) OR (wohlbefinden[Title]) OR (wohlbefinden[MeSH Terms]) OR (stimmung[Title]) OR (stimmung[MeSH Terms]) OR (emotion[Title]) OR (emotion[MeSH Terms]) OR (sentiment[Title]) OR (sentiment[MeSH Terms]) OR (gefühl*[Title]) OR (gefühl*[MeSH Terms]) OR (stress*[Title]) OR (stress*[MeSH Terms]) OR (distress*[Title]) OR (distress*[MeSH Terms])) NOT (marke*) NOT (konsum*) NOT (kauf*) NOT (medika*) NOT (marketing*) NOT (finanz*)

**S1.2 Exclusion and inclusion criteria**

Inclusion Criteria

1. Use of Social Networks as Digital Platforms: The study must focus on social networks as digital platforms that allow users to interact, exchange information, and share content.
2. Relevance to Psychological or Psychosocial Factors: The primary content of the study must involve the identification or assessment of psychological or psychosocial factors, such as behavior, mental health, well-being, or related aspects.
3. Use of Social Media Data: The study must utilize data from social media platforms, defined as computer-based and online technologies that enable social interaction through user-generated content.
4. Language Availability: Articles must be available in either English or German.
5. Study Design: All study designs are eligible; there are no restrictions regarding methodological approaches.
6. Crisis and Disaster Context: The study may focus on any type of crisis or disaster situation but must clearly involve a crisis context.
7. Time Frame: No restrictions apply regarding the publication period; articles from all years may be considered
8. Geographical Scope: Studies from all countries are eligible for inclusion.
9. Peer-Reviewed Scientific Publications: Only peer-reviewed journal or conference publications are included in the analysis.

Exclusion Criteria

1. Definition of Social Networks: Studies that define social networks as primarily informal and enduring relationship structures among individuals or groups (rather than as digital platforms) are excluded.
2. Focus on Political or Marketing Factors: Studies that primarily address political phenomena, marketing strategies, or the influence of social media on political discourse are not included.
3. Social Media as Contact Tool: Studies that use social media merely as a tool for recruiting participants or initiating contact are excluded.
4. Studies Focusing Solely on Social Media Usage Effects: Research that investigates only the general effects of social media use on individuals (e.g., correlations between usage frequency and psychological symptoms) is excluded.
5. Commentaries or Opinion Pieces: Articles that consist solely of comments or opinion pieces on existing studies are not considered.
6. Diagnostic Studies without Crisis Context: Studies analyzing social media for the diagnosis of psychological disorders or suicidal risks without direct reference to a crisis or disaster context are excluded.
7. Organizational Reputation during Crises: Studies focusing on reputational risks of organizations during crises, as reflected on social media, are excluded.
8. Public Health as a Medical Issue: Articles addressing public health strictly as a medical issue, without a psychosocial or psychological focus, are excluded.
9. Reviews Included in Review Comparisons: Review articles that were part of the comparative review overview are not included in the final result synthesis.
10. Individual Psychological Crises without Collective Context: Studies examining individual psychological crises without connection to broader social or collective crisis or disaster events are excluded.
